# Race-based sampling, measurement and monitoring in health data: promising practices to address racial health inequities and their determinants in Black Canadians

**DOI:** 10.24095/hpcdp.45.4.02

**Published:** 2025-04

**Authors:** Margaret Jamieson, Alexandra Blair, Beth Jackson, Arjumand Siddiqi

**Affiliations:** 1 Institute of Health Policy, Management and Evaluation, University of Toronto, Toronto, Ontario, Canada; 2 Social Determinants of Health Division, Public Health Agency of Canada, Ottawa, Ontario, Canada; 3 Dalla Lana School of Public Health, University of Toronto, Toronto, Ontario, Canada

**Keywords:** racism, health surveys, censuses, determinants of health, health equity

## Abstract

**Introduction:**

Racial health inequities are explained by inequities in access to medical advice and treatment, and the physiological effects of inequities in material conditions and everyday life; however, Canadian evidence on racial health inequities is limited. This review describes promising practices in population survey methods and approaches that can strengthen sampling, measurement and monitoring of racial health inequities and determinants of health for population subgroups within Canada—particularly Black Canadians.

**Methods:**

We employed three steps to identify promising practices in Canada’s peer countries and their applicability to the Canadian context. First, we conducted a scan of websites based on prior knowledge of population-level health surveys and datasets. Second, we conducted a review of publications from 2010 to 2020 to identify any missed surveys and datasets. Third, we conducted a targeted review of Canadian population-level health surveys and data sources to identify challenges to and opportunities for implementing these promising practices.

**Results:**

We identified 20 relevant surveys and data sources from the US, the UK, Australia and New Zealand. In several of Canada’s peer countries, information on area-level racial or ethnic concentration of residents is used to conduct targeted sampling strategies, increasing the non-White sample. Our search of the available Canadian datasets found that Canadian health surveys and administrative sources do not routinely incorporate these strategies.

**Conclusion:**

Canada could improve the measurement and monitoring of racial health inequities by applying enhanced sampling practices to collect racial data in surveys and improving procedures for administrative and other routinely collected data sources. There are also novel predictive methods being used to improve sampling of non-White groups, though further investigation of these methods is required.

**Highlights**

- To date, Canadian health surveys have not routinely incorporated strategies to ensure the collection of sufficiently large and representative samples of non-White racialized groups—particularly Black Canadians.
- In several of Canada’s peer countries, information on area-level racial and/or ethnic concentration of residents is used to conduct targeted sampling strategies, thereby increasing the sample of non- White groups.
- Canadian survey sampling strategies could be strengthened by incorporating targeted sampling and oversampling to produce larger and more racially representative samples for Canadian health surveys.
- Novel predictive modelling strategies to collect larger and more representative non-White samples and Canadian efforts to routinely include information on race in administrative data sources should receive particular attention.

## Introduction

Over the last several decades, there have been advances in our understanding of racial health inequities in Canada. However, we still know very little about them, particularly when compared to the United States (US), where much of the evidence on racial health inequities has been produced—particularly on inequities between Black and White Americans. Research into racial health inequities in Canada has been hampered by several factors; notably, the lack of information on race in survey and administrative sources of health data, and insufficiently large or representative samples of non-White groups included in these sources. This review focusses on racial health inequities, a problem that sits at the nexus of three issues: health inequities,^1^ anti-Black and other forms of racism^2^ and concerns about the availability of insightful data about marginalized populations—particularly racialized Canadians.^3^-^5^

There is a well-established body of scientific evidence on racial health inequities—differences in health status across racial groups that are attributable to unjust causes.^1^ This research overwhelmingly comes from the US, and demonstrates that racial health inequities have persistently impacted a wide range of health outcomes across the life course.^6^,7 Compared to Whites, most non-White groups have worse health status, with the most egregious inequities occurring between Whites and Blacks.^6^ The literature demonstrates that these inequities are attributable to racism, the “cultural and structural system that assigns value and grants opportunities and privileges based on race.”^8^^,p.2^ Racism is an organized system that differentially distributes social resources and opportunities based on the hierarchical ranking of racial groups, and includes the concepts of “settler colonialism,”^9^ “racial capitalism”^10^ and “orientalism.”^8^,^11^ These systems shape the distribution of resources, opportunities and power to favour and enable White hegemony, and have all been defined as the supporting “pillars” of White supremacy.^11^,^12^

The history of Black people in Canada—including historically enslaved and not enslaved Black people, and the proportionately larger group of Black immigrants who arrived following the abolition of slavery in the British Empire—is distinct from the history of Black people in the US.^13^-^15^ Consequently, it is critical that Canada understands its own racial health inequities, rather than relying solely on evidence from other countries. Within Canada, health inequities are often most prominently documented between Whites and First Nations, Inuit and Mtis people. The impact of colonialism, anti-Indigenous racism, enslavement and forced assimilation on the health of Indigenous peoples cannot be understated.^16^ Forced displacement, systematic discrimination and the effects of the residential school systems are among other factors that have contributed to consistently poor health outcomes.^17^

Remarkably, we know very little about racial health inequities in key population health benchmarks, such as mortality and life expectancy. Population-level statistical evidence describing how racial health inequities vary by intersecting social positions is sparse in Canada. A major concern is that the best available population-based statistics measuring racial health inequities in Canada are still derived from very small, nonrepresentative samples of non-White groups. The ability to capture specific forms of racism, such as anti-Black racism, in Canada is limited by small samples of Black Canadians—a group representing 4.3% of the Canadian population in the 2021 census.^18^ Our aim in conducting this review is to describe promising practices in population survey methods and approaches that can strengthen the measuring and monitoring of racial health inequities and determinants of health for small population subgroups within Canada—particularly Black Canadians.

## Methods

We employed three key steps of data collection: (1) purposeful sampling of datasets in Canada’s peer countries based on prior knowledge; (2) a scoping review of academic literature in Canada’s peer countries; and (3) a website search of Canadian data sources.

***Step 1: purposeful sampling of datasets based on prior knowledge***

First, we conducted a scan of websites based on our prior knowledge of population-level health datasets. This “purposeful sampling”^19^ strategy to produce a list of data sources was based on existing knowledge of the senior investigator of this paper (AS) gleaned from 20 years of population-level research into health inequities and the social determinants of health in the US and Canada. We restricted our search to data sources that are available in Canada’s “peer” countries, which we defined as the set of countries categorized as “liberal market economies.”^20^,^21^ These countries are all longstanding, high-income democracies that receive large numbers of immigrants from around the world, and have the resources, institutions and autonomy to conduct population health surveys. Aside from Canada, this group of selected countries consists of Australia, New Zealand, the United Kingdom (UK) and the US. 

After identifying our data sources, we conducted a comprehensive website scan for documentation associated with each data source. We created a spreadsheet to record information pertinent to the goals of this review, including information on the types of questions asked, the sample size, the level of subgroups that could be reported upon, and any details or concerns about the validity or reliability of reported measures (available on request from corresponding author). 

Data extraction was conducted by one reviewer (MJ), with validation provided by the other authors (AB, BJ, AS), ensuring all relevant aspects of the data source were captured during extraction. We also produced short, narrative descriptions of this information for each dataset, which are available on request.

***Step 2: scoping review of academic literature***

Next, we conducted a systematic scoping review to capture all the population data sources among Canada’s peer countries that have been used to investigate racial health inequities, and thus supplement our purposeful sampling strategy. One author (MJ) drew up preliminary drafts of search criteria, with regular validation from the other authors (AB, BJ, AS). A librarian at the University of Toronto’s Gerstein Science Information Centre provided additional consultation and advice. To streamline our search process, we restricted our query to a single database. Our primary objective was to uncover any potentially missed health datasets rather than to compile a comprehensive list of all studies utilizing population-level health data. In consultation with the librarian, we concluded that conducting additional searches in other databases would be redundant. These searches were unlikely to reveal datasets beyond those already identified through our chosen database (MEDLINE) and might instead retrieve datasets not related to health, which would fall outside the scope of our investigation. The final search criteria for MEDLINE are shown in Table 1.

**Table 1:** Search strategy for systematic scoping review

Theme (in title, abstract or keywords)	Search terms
Health record or registry-based data	((((record* or certificate* or registries or registry) adj (medical or birth or death or dental or hospital or nursing)).ab,kf,ti. OR exp Records/)
Survey-based data	((survey* or questionnaire* or census).ti. OR exp “Surveys and Questionnaires”/)))
Race- or ethnic group-based data	(exp “Ethnic Groups”/ OR exp Population Groups OR (race or races or racis* or racial or ethnic* or ethno*).ab,ti,kf. OR ((african adj3 american*) or (black adj2 (individual* or person* or people*))).ab,ti,kf.)
MEDLINE	((((record* or certificate* or registries or registry) adj (medical or birth or death or dental or hospital or nursing)).ab,kf,ti. OR exp Records/) AND ((survey* or questionnaire* or census).ti. OR exp “Surveys and Questionnaires”/))) AND (exp “Ethnic Groups”/ OR exp Population Groups OR (race or races or racis* or racial or ethnic* or ethno*).ab,ti,kf. OR ((african adj3 american*) or (black adj2 (individual* or person* or people*))).ab,ti,kf.)

Results were limited to English language, human research subjects and publication between 2010 and 2020. Papers went through title and abstract screening (MJ, AS), followed by full-text screening (MJ, AS) in which the following exclusion criteria were applied:

- Duplicate papers
- Full paper not available
- Not an English language publication
- Not conducted in a Canada-peer country
- Does not measure race or ethnicity, or is not chiefly focussed on race
- Data source is neither a survey nor an administrative data source (i.e. data source is a cohort study, or a non–population level registry that was not linked to a larger dataset)
- Data source described is not focussed on health
- Data source described is focussed on the delivery of clinical care
- Data source is a subnational cancer registry
- Study described sampled populations from within the described dataset sample
- Opinion piece or commentary

**Figure 1:**
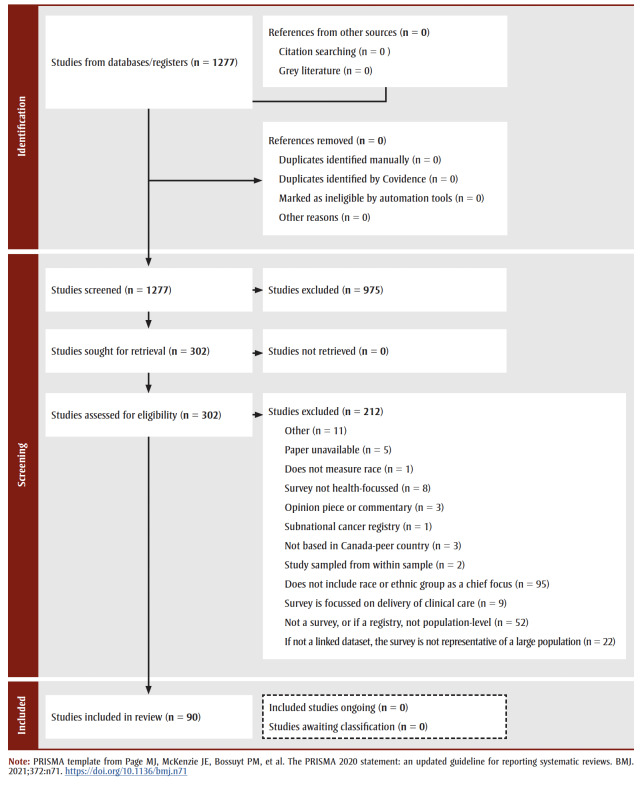
PRISMA diagram

Some papers were excluded if they used surveys that were technically related to “health,” but focussed more specifically upon healthcare or healthcare workers (as in the Nurses’ Health Study), or upon diet (as in the UK’s National Diet and Nutrition Survey). Covidence software22 was used for the screening stage of the scoping review, followed by data extraction (MJ, AS) to obtain the same parameters of information as yielded in the initial website scan. The final PRISMA diagram for the scoping review is shown in Figure 1. 

Because the goal of this scoping review was to glean information about available data sources in the literature, we sorted included papers into five general categories throughout the screening and extraction process:

- 1. Papers using large survey datasets not otherwise identified in Step 1
- 2. Papers using survey datasets that we had already identified in Step 1
- 3. Papers using survey datasets that have been linked to administrative datasets, other large surveys, census data or other data sources that are nationally representative
- 4. Papers using population-level cancer datasets focussing on race and ethnicity
- 5. Papers about survey and data linkage methods focussing on race and ethnicity

Given that the focus of this review was to learn more about the datasets themselves, there was no rigorous data extraction of these papers, and they have not been cited here (details are available upon request). Having now identified gaps in our sample of relevant surveys and datasets, we consulted available websites from data stewards to obtain the relevant information on sampling procedures and other details about the surveys and data sources. 

***Step 3: website search of Canadian data sources***

Finally, we sought to identify the relevant sources of Canadian data, conducting a focussed search of Statistics Canada’s websites, as well as the websites of data stewards in Ontario, British Columbia and Manitoba. Included data sources were limited to those that included questions (in the case of surveys) and reporting variables (in the case of the administrative data products) on health. For the surveys, we chiefly identified these first by filtering Statistics Canada’s list of surveys and statistical programs by keyword,^23^ then by consulting the questionnaires themselves. Although our main interest was in surveys or sources that were still being actively updated (such as the census) to inform future practice, we also included sources for which data collection had ended (such as the LSIC*). These were deemed relevant because they could still be actively used by Canadian researchers to answer health-related questions, and therefore their data collection biases could impact findings. Surveys that only contained information on activity limitations, but no other health questions, were not included. 

Information on all data sources was reviewed in 2024, to capture any changes to methodologies that might have occurred since initial data collection and abstraction.

*See Table 2 for full names of surveys.

## Results

***Peer country datasets***

In total, 20 international datasets were identified through Steps 1 and 2. In Step1, the purposeful sampling procedure yielded the 11 surveys or administrative sources of data listed in Table 2 and described in Table 3. A comprehensive summary of the data sources is available from the authors upon request. Of these 11 data sources, nine were based in the US and two in the UK. Nine data sources were national population-based surveys (BRFSS, HRS, NHANES, NHIS, Add Health, NSAL, PRAMS, BHPS and HSE), while two sources (CDC WONDER and HIP) were based on administrative sources of data.

**Table 2 a:** Data sources from Canada and peer countries identified in Steps 1 to 3 Peer country datasets identified in Step 1 (purposeful sampling)

Dataset	Country
Behavioural Risk Factor Surveillance Survey (BRFSS)^24^	US
British Household Panel Survey (BHPS)^25^	UK
CDC WONDER^26^	US
Health and Retirement Study (HRS)^27^	US
Health Inequality Project (HIP)^28^	US
Health Survey for England (HSE)^29^	UK
The National Health and Nutrition Examination Survey (NHANES)^30^	US
The National Health Interview Survey (NHIS)^31^	US
National Longitudinal Survey of Adolescent Health (Add Health)^32^	US
National Survey of American Life (NSAL)^33^	US
Pregnancy Risk Assessment Monitoring System (PRAMS)^34^	US

**Table 2b:** Data sources from Canada and peer countries identified in Steps 1 to 3 Peer country datasets identified in Step 2 (systematic scoping review)

Dataset	Country
California Health Interview Survey (CHIS)^35^	US
Mortality Disparities in American Communities Survey (MDAC)^36^	US
Midlife in the United States (MIDUS)^37^	US
New South Wales Population Health Surveys (NSWPHS)^38^	Australia
New York City Community Health Survey (NYCCHS)^39^	US
New Zealand Health Index and Associated Population Health Registries (NHI)^40^	New Zealand
New Zealand Health Survey (NZHS)^41^	New Zealand
Surveillance, Epidemiology and End Results (SEER)^42^	US
Understanding Society: The United Kingdom Household Longitudinal Study^43^	UK

**Table 2c:** Data sources from Canada and peer countries identified in Steps 1 to 3 Canadian datasets identified in Step 3 (website search)

Dataset	Country
Canadian Community Health Survey (CCHS)^44^	Statistics Canada
Canadian Health Measures Survey (CHMS)^45^	Statistics Canada
Canadian Health Survey on Children and Youth (CHSCY)^46^	Statistics Canada
Canadian Health Survey on Seniors (CHSS)^47^	Statistics Canada
Canadian Housing Survey (CHS)^48^	Statistics Canada
Canadian Survey on Disability (CSD)^49^	Statistics Canada
Census-linked health records^50^	Statistics Canada
Hospital Mental Health Database Metadata (HMHDB)^51^	Canadian Institute for Health Information (CIHI)
Labour Force Survey (LFS)^52^	Statistics Canada
ICES (formerly Institute for Clinical and Evaluative Sciences)^53^	ICES
Manitoba Population Research Data Repository (MPRDR)^54^	MPRDR
Longitudinal Survey of Immigrants to Canada (LSIC)^55^	Statistics Canada
National Population Health Survey (NPHS)^56^	Statistics Canada
National Longitudinal Survey of Children and Youth (NLSCY)^57^	Statistics Canada
Population Data British Columbia (PopData BC)^58^	PopData BC

**Abbreviations:** UK, United Kingdom; US, United States. 

**Table 3:** Descriptions of health surveys and administrative data sources in Canada and Canada’s peer countries

Name and country/area of dataset	Years of data availability	Target population	Sampling frame	Linkage, sampling strategy
Behavioral Risk Factor Surveillance Survey (BRFSS; US)	Information presented as of 2023 cycle, but survey dates to 1985	Adult US residents	Adults living in households, excluding vacation homes, group homes and institutions	State-specific sampling strategy based on landline and cellular telephone numbers. The landline strategy first divides each state into blocks (e.g. streets) within each state into high-density and low-density strata. This is somewhat analogous to a “probability proportional to size” method of sampling, which is the primary strategy for surveys such as NHIS and NHANES. Since 2017, 11 states have also engaged in oversampling strategies, to obtain larger samples of American Indians^b^ and Alaskan Natives.^b^ Each state may also have additional oversamples (by race, region and so on).
British Household Panel Survey (BHPS; UK)	Information presented as of latest wave prior to merging with *Understanding Society* in 2018 cycle, but survey dates to 1991	Population of the UK	Households sampled in 1991	The initial sample of 5500 households was obtained through a stratified, clustered design that drew on the Postcode Address File. Additional samples from Scotland, Wales and Northern Ireland were also added.
Health and Retirement Study (HRS; US)	Information presented as of 2020, but survey dates to 1992	Older adults in the US	Residents of the contiguous US, aged 51–61 at the collection of each wave, following through to subsequent waves; excludes institutionalized populations	Linked to US death records. HRS uses a multistage, area-level probability sample of US households. In the first stage, US geographic regions (e.g. cities, counties) are selected using probability-proportionate-to-size logic. In the second stage, area segments are sampled within these regions. Finally, housing units, and then “household financial units” within household units are selected. HRS includes oversamples of Blacks, Hispanics^b^ and residents of Florida. For example, based on 1990 census data, census block groups containing more than 10% of Black-led households were located and placed in a separate stratum in the second stage of sampling. This stratum was oversampled, and thus people in this stratum had a higher probability of being selected for the sample. This strategy increased the Black sample from 10% to 18.6% of the overall HRS sample.
Midlife in the United States (MIDUS; US)	Information presented as of the latest cycle in 2014, but first survey dates to 1995/96	Americans in mid-life	All noninstitutionalized, English-speaking adults, 25–74 years of age, in the contiguous US	The baseline sample consisted of four subsamples: (1) a national random-digit dialling sample (n = 3487); (2) siblings of individuals from the national random sample (n = 950); (3) oversamples from five metropolitan areas in the US (n = 757); and (4) a national random-digit dialling sample of twin pairs (n = 1914).
National Survey of American Life (NSAL; US)	Survey conducted between 2001 and 2003	Population of Black and White persons in the US	Sampling based on a national household probability sample	Sampling was based on area-level density of African-American and Afro-Caribbean people, and Whites were selected from the same geographic areas, so that racial groups represented in the survey had similar contextual and geographic characteristics.
New Zealand Health Survey (NZHS) (New Zealand)	Information presented as of 2023/24 cycle, but survey dates to 1992/93	“Usual resident” population of New Zealand of all ages, including those who live in private dwellings	Population excluding residents of nonprivate dwellings, foreign diplomats, individuals located on islands other than the North, South and Waiheke islands	Information can be linked to other sources from the New Zealand government, asks for consent to link data. No explicit information on linkage is provided, but respondents are asked to provide their date of birth, address and name for linkage. Uses a multistage, stratified, probability-proportional-to-size sampling design, intended to sample 14 000 adults and 5000 children yearly. Uses a dual-frame approach in which respondents are selected from an area-based sample and a list-based electoral roll sample, with the aim of increasing sample sizes for Māori and other ethnic groups. NZHS uses two sampling strategies, done explicitly to increase the number of specific ethnic groups (Māori, Pacific and Asian respondents) in the sample. The first strategy uses clusters of blocks as primary sampling units, and conducts a multistage, probability-proportional-to-size method to select households. Block clusters are also differentiated by the proportion of Pacific and Asian residents, also done to increase the sample size of these groups. The second strategy uses New Zealand electoral rolls, in which Māori ethnicity is self-identified, to increase the sample of Māori. The electoral rolls are used to locate block clusters that have a higher proportion of Māori residents, and then households from these block clusters are differentially selected.
Health Survey for England (HSE; England)	Information presented as of 2022 cycle, but survey dates to 1991	Population of private households in England	Households in England, excluding institutional population	Information from some health and medical records could be added to the responses given in the survey, asks consent for this information. Survey uses name, address and date of birth to identify health records before they are linked to the anonymized survey data. Links hospital episode statistics data, mortality data, cancer registration data. Survey used a stratified random probability sample of households. 2018 survey oversampled the North East and East Midlands regions to ensure a minimum sample size of ~700 adults. In some years, the core sample is augmented to boost the number of responses from certain populations such as ethnic groups, older adults or children; however, no such boost was done to the 2022 survey. In 2004, the HSE focussed on the health of ethnic minorities, in which they obtained “boost samples” (i.e. oversamples) of several non-White racial and ethnic groups. These oversamples were based on targeting of areas in which these groups were in high proportions.
National Longitudinal Survey of Adolescent Health (Add Health; US)	Fifth wave of data collection presented (2016–2019), but earliest survey wave dates to 1994	Adolescents in the US and their parents and partners (in some waves only)	Adolescents sampled from American high schools in Grades 7–12 in 1994/95	Data for individual respondents linkable across other waves of the survey. Initial wave composed of two samples: a school sample and an in-home sample. The school sample was collected through a stratified, random sample of all high schools in the US. Stratifying variables included region, urbanicity, school size, school type (public, private, parochial), percent White, percent Black, grade span and curriculum (general, vocational, etc.). The in-home sample consisted of a core sample from each community plus some oversamples. Contains an oversample of Black individuals with at least one parent who has a college (undergraduate) degree, which ensures broad socioeconomic representation of Black people in the sample. Similarly, Add Health also includes oversamples of Asians, Cubans and Puerto Ricans to ensure their representation in the sample. The oversamples were obtained through individuals’ responses on school questionnaires.
National Health Interview Survey (NHIS; adults, US)	Information presented as of 2023 cycle, but survey dates to 1957	Adults in noninstitutional households	Households in the US	Linkage done to other data sources using last 4 digits of social security number or Medicare number. Cross-sectional household interview survey, with sampling and interviewing occurring continuously throughout the year. Sample strategy is redesigned every 10 years. The total sample is subdivided into 4 separate panels, such that each panel is representative of the US population. The probability of an area being selected is not equal. Rather, areas are stratified by sociodemographic characteristics, and the probability of an individual being selected is higher in some strata than others. This methodology of “oversampling” for some characteristics ensures that the survey will have sufficient representation of these characteristics to enable robust analyses of them. Thus, until 2016, NHIS contained oversamples of several racial and ethnic groups (Blacks, Hispanics,^b^ Asians were all oversampled by 2006). However, in the 2016 redesign, there are no longer oversamples of racial groups. A new sample design based on the 2020 census will be introduced in this NHIS in 2025, though details have not yet been revealed.
National Health Interview Survey (NHIS; children, US)	Information presented as of 2023 cycle, but child component of survey dates to 1997	Children under 17 y in noninstitutional households	See NHIS (adult) entry, above	See NHIS (adult) entry, above
National Health and Nutrition Examination Survey (NHANES; US)	Information presented as of 2017–2020 cycle, but survey dates to 1971	National, noninstitutional residential population	Noninstitutionalized civilian population residing in the 50 states and the District of Columbia	Linkage using social security number provided during survey, when linkage is performed. Multiyear, stratified, clustered 4-stage sample, with sampling units and their tiers identified under “sampling unit.” Oversamples are produced for several racial and ethnic groups, including Blacks, Hispanics^b^ and Asians. Oversamples are also produced for all those who fall below 185% of the US poverty threshold, and for those at either end of the age distribution. These oversamples are produced by using US census data to locate areas with higher proportions of individuals with these characteristics. Nonetheless, there is some data to indicate poorer people and Black people may be more likely to be missing from the sample, and more likely to have missing data in the survey.
New South Wales Population Health Surveys (NSWPHS; Australia, NSW)	Information presented as of 2023 survey	Adults and children of NSW	Varies by survey	Random-digit dialling of both landline and mobile phone users. No further information is found about sample design for the adult survey, although the school student behaviour survey samples schools. Publicly available methodological documentation has not been updated since 2011.
California Health Interview Survey (CHIS; US, California)	Information presented as of 2023 cycle, but earliest information available as of 2001	Population of California	Sample of California addresses, with initial communication about the survey completed with a letter to the address, followed up with a phone call	Prior to 2019, CHIS used a telephone-based sampling frame. In 2019, CHIS converted to an area-based sampling frame, with households stratified by area (i.e. counties) in ways that capture larger racial and ethnic groups in California. Uses a novel predictive modelling methodology to obtain samples of smaller racial and ethnic (and other sociodemographic) subgroups that are more difficult to capture through area-based sampling. To do so, the previous year’s CHIS was linked to several other sources of data, including voter registration databases and consumer databases, census planning data. Random forest models were run to predict households that had residents who are Filipino, Vietnamese and so on. Households that had residents with these characteristics were placed in a separate stratum, and residences and then individuals were randomly selected within this stratum. Additionally, in 2023 the survey introduced an oversample of numbers associated with pre-paid cell phones, which are more likely to be used by Hispanics,^b^ people with lower education and lower income, and other underrepresented groups.
New York City Community Health Survey (NYCCHS; US, New York City)	Information presented as of 2020 cycle, but survey dates to 2002	Adult population of New York City, living in nongroup settings	Adults aged 18+ living in the 5 boroughs of New York City, with access to either a cell phone or landline	Stratified random sample based on neighbourhood of inhabitant (ascertained using zip codes). Ten thousand households are randomly selected from a sample of phone numbers that are stratified by neighbourhood.
Understanding Society (UK)	Information presented as of 2021–2023 cycle, but survey dates to 2009	General population aged 10+ of the UK	Households in Great Britain and Northern Ireland	Asks for consent to link responses to government records, including health records. The sampling strategy contains multiple components. The General Population Sample (approximately 26 000 households) is a clustered and stratified probability sample of households in Great Britain and a simple random sample of households in Northern Ireland. The survey also contains an ethnic minority boost sample (approximately 4000 households) selected from areas of high ethnic minority concentration in 2009–2010 where at least one member was from an ethnic minority group. An immigrant and ethnic minority boost sample (approximately 2900 households) was added in Wave 6 (2015) of the study, and it was also obtained from areas of high ethnic minority concentration in that year. Finally, a sample from the BHPS (approximately 8000 households) was added in Wave 2.
Pregnancy Risk Assessment Monitoring System (PRAMS; US)	Information presented as of 2023, but surveillance system dates to 1987	Pregnant women and new mothers	Birth certificate files from each state	Birth certificate and infant death certificate (when applicable) can be used for linkage. Some states also link to Medicaid data and other records. PRAMS samples women who have had a recent live birth, and the sampling frame is the birth certificate file of each state. Each state samples 1300–3400 women per year. Women from some groups (including Black and other racialized groups) are sampled at a higher rate to ensure their representation. The states that sample by race are Alaska, Connecticut, Florida, Iowa, Louisiana, Massachusetts, Michigan, Minnesota, Nebraska, New Jersey, New Mexico, Oregon, Texas, Washington, Wisconsin, Wyoming. Publicly available methodological documentation has not been updated since 2018.
Canadian Community Health Survey (CCHS; Canada)	Information presented as of 2024 cycle, but survey dates to 2001	Canadian population aged 12+ y	Population aged 12+, excluding persons living on reserves and other Aboriginal^b^ settlements in the provinces; full-time members of the Canadian Forces; the institutionalized population, children aged 12–17 y that are living in foster care, and persons living in the Quebec health regions of Rgion du Nunavik and Rgion des Terres-Cries-de-la-Baie-James.	Data from 2019 survey were linked to tax records of the participants and all members of the participants’ households. Variables used for linkage included household information (address, etc.), personal information (social insurance number, surname, age, etc.), and information about household members. The survey uses two main sampling frames: an area Canada frame, and a Canada Child Benefit (CCB) frame for the child sample. The CCHS area frame is designed to serve the Labour Force Survey (LFS). LFS first clusters areas, and the clusters are chosen based on probability proportional to size. CCHS places these clusters into health regions, so that sampling of dwellings from clusters assures representation at the health region level. Within each cluster, dwellings are chosen systematically. For the CCB frame, children are selected by simple random sample.
Canadian Health Survey on Seniors (CHSS; Canada)	Information presented as of most recent 2020 cycle, but the survey dates to 2018	Canadian population aged 65+ y	The target population is individuals aged 65+ y living in Canada’s 10 provinces. Exclusions include people living on reserves and other Aboriginal^b^ settlements, full-time members of the Canadian Forces, institutionalized populations and persons living in certain regions of Quebec.	Asks for permission to link the responses of the survey to the CCHS and tax data, and reminds participants that their responses may be linked by their provincial governments to other data sources. The sampling design includes selecting those aged 65 y or older who are included in the CCHS, and an oversample in all provinces except Ontario and Quebec (where no oversample was required to read sample target numbers). The oversample obtained through random selection was a sampling frame of all telephone numbers for all households with at least one occupant aged 65 y or older.
Labour Force Survey (LFS; Canada)	Information presented as of 2024 cycles, but latest re-design was implemented in 2007	Noninstitutionalized population of Canada aged 15+	Exclusions from the target population are persons living on reserves and other Aboriginal^b^ settlements in the provinces, full-time members of Canadian armed forces, institutionalized populations and households in extremely remote areas with very low population density.	LFS uses a stratified, multistage design. Each province is considered a unit. Within each province, smaller geographic areas (clusters) are selected, and from each cluster, households are selected. LFS uses a rotating panel sample design so that selected households remain in the LFS sample for six consecutive months. Each month, one-sixth of the LFS sample is in their first month of the survey. One feature of the LFS sample design is that each of the six rotation groups can be used as a representative sample by itself.
Longitudinal Survey of Immigrants to Canada (LSIC; Canada)	Information presented from first survey conducted in 2001, study later concluded in 2005	The target population consists of immigrants who: arrived in Canada in 2000/01, were aged 15 y or older at the time of landing and landed from abroad, having applied through a Canadian Mission Abroad	Individuals who applied and landed from within Canada were excluded from the survey as they may have been in Canada for considerably longer, and therefore may demonstrate different integration characteristics than those more recently arrived. Refugees claiming asylum from within Canada were also excluded from the survey.	Statistics Canada’s website does not list any data linkages, although the Canadian Research Data Centre Network says the survey data has been linked to the Canadian Vital Statistics – Death Database and the Canadian Cancer Registry. The sample was made up of 12 cohorts, or, 12 independent monthly samples. The sampling frame was the administrative database of all landed immigrants to Canada that comes from Immigration, Refugee and Citizenship Canada (formerly Citizenship and Immigration Canada). The sample was first stratified by month of landing, intended province of destination and class of immigrant. The first stage of sampling involved selecting Immigrating Units (which refers to people who apply under the same visa form) within each stratum using a probability-proportional-to-size method. The second stage involved selection of one or more respondents within each Immigrating Unit. The initial sample consisted of 12 000 individuals, which declined to 7700 individuals by the third (and final) wave. Does not seem to have been any sampling by race, although there are groups that have been oversampled throughout the different waves of the survey, including (1) government sponsored refugees; (2) refugees other than government-sponsored; (3) contractor and investor immigrants (economic-business); (4) family immigrants in British Columbia; (5) overall immigrants in Alberta; and (6) economic immigrants in Quebec (economic-skilled and economic-business).
Longitudinal and International Survey of Adults (LISA; Canada)	First wave conducted 2012, latest wave in 2020	Canadians aged 15+ y. The target population excludes those living in Canada’s territories, those living on reserves or other Aboriginal^b^ settlements in the provinces, official representatives of foreign countries living in Canada and their families; members of religious and other communal colonies; members of the Canadian Armed Forces stationed outside of Canada; those living full-time in institutions, e.g. inmates of correctional facilities and chronic care patients living in hospitals and nursing homes; and those living in other collective dwellings.	The LISA sampling frame includes all households from the 2011 census that were not eligible for the National Household Survey, which was conducted at the same time.	Because part of LISA was integrated with part of the Programme for the International Assessment of Adult Competencies, also known as the International Study of Adults (ISA), households were stratified into ISA and non-ISA samples. Within each of these strata, households were further stratified by province and urban or nonurban status. In the stratum ineligible for ISA, the provincial and urban/nonurban stratification and the geographic clustering were identical to that described above. The selection of dwellings in this stratum, however, was done in only one phase using simple systematic sampling. Again, all members of the households in the selected dwellings became members of the LISA sample and formed the LISA-only sample. For the ISA stratum, there was further consideration of probability proportion to age (15–65 y).
National Longitudinal Survey of Children and Youth (NLSCY; Canada)	Information presented as of latest cycle in 2008/09, but survey dates to cycle 1 in 1994/95	Noninstitutionalized civilian population from Canada’s 10 provinces who are aged 0 to 11 y at the time of selection into the study	Exclusions include children living on Indian^b^ reserves or Crown lands, residents of institutions, full-time members of the Canadian Armed Forces and residents of some remote regions.	Ability to link is unknown, although the datasets are stored within Statistics Canada’s social data linkage environment, suggesting it can be linked to other data sources. The questionnaire booklet only mentions combining data from the given survey year with data from other years, however. NLSCY consists of both longitudinal and cross-sectional samples. Each longitudinal sample is representative of the original (1994) longitudinal sample. Cross-sectional weights are provided that allow age cohorts to be considered representative of a cross-sectional population of a given age group. All NLSCY samples have been drawn from the sample of LFS respondent households (with cycle 1 also drawn from the NPHS sample).
Canadian Health Measures Survey (CHMS; Canada)	Information presented as of 2022–2024 cycle, but survey dates to cycle 1 in 2007–2009	Population of Canada aged 3–79 y	Persons aged 3 to 79 y living in the 10 Canadian provinces. The target sample thus excludes those individuals living in the three Canadian territories, people living in institutions in Canada and those in the Canadian Armed Forces.	Data from household survey linked (with permission) to tax records. Individual clinic data linked (with permission) to provincial-level health information using personal health number. The primary sampling unit of the CHMS is the “collection site,” which is a geographic unit of about a 50 km radius in urban areas and a 75 km radius in rural areas. Collection sites are stratified by region (British Columbia, Prairies, Ontario, Quebec, Atlantic), and metropolitan region (or other). Within each stratum, dwellings are stratified by age of residents, and broad age category stratification is used to select an individual within a dwelling.
National Population Health Survey (NPHS; Canada)	Information presented as of the latest cycle in 2011, but survey dates to 1994/95	Household residents of the 10 Canadian provinces in 1994/95	Excluded are persons living on Indian^b^ reserves and Crown lands, residents of health institutions, full-time members of the Canadian Armed Forces and persons living in some remote areas in Ontario and Quebec.	Survey data can be linked to provincial health information using personal health number, with follow-up surveys asking if the person’s health number has changed, to make sure records are kept up to date. The NPHS used the LFS as its sampling frame, with the exclusion of the sample from Quebec, which used the Sant Qubec’s design for the 1992/93 Enqute sociale et de sant. The LFS design is detailed elsewhere in this report. In brief, LFS sampling strategy relies on clustering geographic areas. The Quebec sample was similarly geographically clustered. In the first cycle of the NPHS (1994/95), households were randomly selected and individuals within households were selected. The initial sample contained 17 726 individuals.
Canadian Survey on Disability (CSD; Canada)	Information presented as of 2017 cycle, but survey dates to 2012, prior to which it was known as the "Participation and Activity Limitation Survey” and earlier still known as "Health and Activity Limitation Survey (Household Component)"	Canadian youth and adults who are facing long-term conditions or health-related problems	All persons aged 15+ y, and who report having difficulty “sometimes,” “often” or “always” to one of the activities of daily living questions on the 2016 Census of Population long form. The long form census includes persons living in private dwellings in Canada. Persons living on First Nations reserves and in institutions are excluded.	Responses linked to census, particularly to fill in certain demographic information. Approximately one out of every four households is selected for the long form census. Respondents were stratified to assure appropriate estimation of various age groups, residence in remote versus non-remote areas and severity of disability.
Canadian Housing Survey (CHS; Canada)	Information presented as of 2022 cycle, but survey dates to first survey in 2018	Population of Canada	Population of Canada’s 10 provinces and 3 territories, excluding: residents of institutions (including people living in residences for dependent seniors and people living in school residences), members of the Canadian Armed Forces living in military camps and people living on First Nations reserves.	Data linked to tax, income and immigration data by Statistics Canada, as well as potentially to other sources. The sampling frame is the Dwelling Universe File, which is stratified into 43 geographic strata, including the largest census metropolitan area in each province. Each geographic stratum is further stratified between Social and Affordable Housing (SAH) dwellings and all other dwellings. This enables oversampling of those in SAH dwellings. Within each geographic and SAH/non-SAH stratum, dwellings are sorted by predicted household income, and then a systematic random sample is taken to ensure representation of household income.
Canadian Health Survey on Children and Youth (CHSCY; Canada)	Information presented as of 2023 cycle, following the pilot in 2016, and the follow-up in 2017	Children aged 1–17 y in Canada	Children aged 1–17 y in Canada, excluding children living on First Nation reserves and other Aboriginal^b^ settlements in provinces, as well as children living in foster homes and other institutionalized children.	With permission, data are linked to household-level tax records, data from other surveys, and provincial health services records (using personal health number). The sampling frame is children who received the Canada Child Benefit (CCB), which, according to 2018 population estimates, covers 98% of the Canadian population aged 1 to 17 y in Canadian provinces, and 96% of this population in the Canadian territories. The CCB file was stratified by province, with territories clustered into a single stratum. In Ontario, further stratification was done by Local Health Integration Network. The sample was then further stratified into three age groups (1–4 y, 5–11 y and 12–17 y). Within these geographic and age strata, children were randomly selected, resulting in a total sample size of 92 170 children.

**Abbreviations:** UK, United Kingdom; US, United States; y, years. ^a^ Additional information available from the authors upon request. ^b^ Terminology used in the original survey. 

The scoping review (Step 2) yielded nine additional data sources from 89 papers (Figure 1). Although many papers in the scoping review identified datasets of potential interest, only nine were included for this paper. These additional sources are listed in Table 2, and described in Table 3. Of these data sources, five sources were based in the US, two were from New Zealand, one was from Australia and one was from the UK. Five sources were population-based health surveys (CHIS, MIDUS, NSWPHS, NZHS and Understanding Society), while three sources (MDAC, SEER and the NHI) were based on administrative sources of data. All sources contain questions about race or ethnicity, except for the NSWPHS, where it is somewhat unclear.

***Canadian datasets***

Our search of the Canadian information produced 15 data sources, listed in Table 2 and described in Table 3. All of the surveys contain questions about race or ethnicity, but the administrative data sources generally did not include this information. Table 2 lists these sources, which are described in Table 3. 

***Characteristics of peer country surveys***

For each survey, the target population was all individuals living in households in the country (or state or city in a few cases) in which the survey was being conducted. Some surveys had target populations that excluded some regions or areas. This approach typically excludes people living in institutions, such as prisons or nursing homes, and people experiencing homelessness or precarious housing. 

The sampling frame used to obtain the set of households was generally composed of administrative records containing all addresses or telephone numbers of residents in the geographic area. Addresses and telephone numbers were then generally stratified or clustered by various characteristics (including location), then randomly selecting households within each stratum or cluster and finally selecting individuals within each household. One survey (NSWPHS) contained very little information about its sampling strategy. It appeared to use a telephone-based sampling frame, and obtained its sample from random-digit dialling, but it is unclear whether any intermediate stratification steps were taken. 

All US-based surveys explicitly incorporated race or ethnicity into their sampling strategy. Usually, this involved creating strata based on census estimates of the racial compositions of areas, and oversampling from strata with higher proportions of Blacks and other non-White groups. This was applied by the BRFSS, HRS, MIDUS, NHANES, NHIS and NSAL. The US Add Health surveys used indicators of the proportion of White and Black students in each school to sufficiently sample from schools with higher proportions of Black students. 

An area-based sampling approach to obtain racial representation was also used by the UK’s Understanding Society survey. By contrast, other UK surveys (HSE and BHPS) did not include race in their main sampling strategies. However, in some survey years, such as 2004, the HSE incorporated a “boost sample” of non-White people, which it obtained from census-based area-level information to locate places in which high proportions of people of non-White ethnicity reside.^59^

PRAMS used administrative records that contain individual-level race and/or ethnicity information as a sampling frame from which to collect an oversample of non-White racial and/or ethnic groups. PRAMS samples entirely from US birth records, which contain mother’s (and sometimes father’s) race and/or ethnicity. PRAMS uses the latter information to ensure a sufficient sample of Black and other racialized groups. New Zealand’s NZHS also used a source of administrative data as part of its sampling frame, to ensure a sufficient sample of Māori. NZHS also uses census information to locate areas where Māori, Asian New Zealanders and other non-White ethnic groups reside, to ensure they are included in the sample. 

California’s CHIS is unique for having a very specific mandate to ensure that it collects ample information on the wide range of racial and/or ethnic groups that make up the population of California. Before 2019, CHIS used a telephone-based sampling frame. In 2019, CHIS converted to an area-based sampling frame, with households stratified by area in ways that capture larger racialized or ethnic groups in California. Additionally, CHIS used a novel predictive modelling methodology to obtain samples of smaller racialized or ethnic (and other sociodemographic) subgroups that are more difficult to capture through area-based sampling. 

***Characteristics of peer country administrative sources***

The main source of administrative data in the US that can be used to measure racial and ethnic health inequities is CDC Wonder. This database is hosted by the Centers for Disease Control and Prevention, and contains approximately 11 administrative sources, including all US births and deaths. In some cases, these data are available at the individual level and contain information on individual race and/or ethnicity. In other cases, such as death records, data are available at the area level. Moreover, these county-level data can be readily linked to other data (e.g. census data), which provides county-level information on a variety of social and economic factors. The US-based HIP is a newer source of data that is not hosted by a government entity. HIP links individual-level federal tax return data, social security administrative data and death records in the US between 1999 and 2014. It provides individual-level socioeconomic and demographic characteristics of individuals who died over this period. 

SEER is a US cancer registry, though not all states are included. Some SEER data have been linked to data from Medicare, which is the healthcare insurance system for adults aged 65 years and older. Despite being a census of cancer cases for participating states, analysis of racial inequities using SEER data may still suffer from statistical power considerations, due to issues related to the combination of small population sizes (Asian-Americans are given as an example in the documentation) and rare cancers.

New Zealand mandates collection of ethnicity data in its health and disability sector. Each individual who uses health or disability services has a health number, known as the National Health Index (NHI).^60^ The establishment of the NHI is accompanied by information on the individual’s address, gender and ethnicity. Moreover, other administrative health databases also collect ethnicity data, or can be linked to the NHI. In 2009, an algorithm effectively searched across databases to fill in ethnicity data back to 1989.

***Characteristics of Canadian survey sources***

Most Canadian health surveys have a target population containing all or virtually all households in Canada. Like US surveys with analogous target populations, this excludes Canadians in institutional facilities, such as prisons and nursing homes, and children living in foster care. Other excluded groups include persons living on reserves and other Indigenous settlements in the provinces; full-time members of the Canadian Armed Forces; and persons living in the Quebec health regions of Rgion du Nunavik and Rgion des Terres-Cries-de-la-Baie-James. Other groups that are excluded are persons living in Nunavut outside of the 10 largest communities. Several surveys have sampling frames of all households, with multistage, stratified sampling of these sampling frames producing geographically representative samples. 

Some surveys also use administrative databases for sampling frames. The CHSCY and the child sample of the CCHS use the Canada Child Benefit database as a sampling frame, which is also stratified by geographic regions. It also incorporates age strata into its sampling strategy. The CSD samples people who reported having activities of daily living limitations on the long-form census in 2016 (for the 2017 CSD) and 2011 (for the 2012 CSD). The LSIC uses the landed immigrant registry as its sampling frame.

***Characteristics of Canadian administrative data sources***

Two of the administrative sources were national, while three were provincial health data holdings (British Columbia, Manitoba and Ontario). Statistics Canada has linked the long-form census (which contains information on race and ethnic origin and an extensive set of other sociodemographic and socioeconomic characteristics) to several administrative health records, including births, deaths and hospitalization information, and the Discharge Abstracts Database (DAD). The number of long-form censuses linked to administrative health records varies by health record. The long-form census samples 25% of Canadian households, with the rest receiving the short-form census (which does not query race and/or ethnicity).

At least three provinces have their own administrative data holdings. The most extensive of these appears to be Ontario’s holding, named ICES. The main database in ICES is the medical billing and pharmaceutical records data for the province, containing every physician or hospital encounter, and every drug prescription for the province’s population. ICES has been linked to several other data sources, though not every individual is covered. At the individual level, there are at least three sources. First, ICES has racial and/or ethnic data through its linkage with the CCHS. Second, an algorithm based on last names that aims to identify people who are South Asian- or Chinese-Ontarians was integrated in ICES. Finally, ICES has a linkage to the immigration administrative database for Ontario that contains information on country of origin, which in many cases can be used as a proxy for race and/or ethnicity. At the area level, ICES is linked to the census, and so area-level ethnic concentration is also available. 

PopData BC appears to have fewer racial and/or ethnic data linkages, though again, it was difficult to assess their complete holdings. It appears that PopData also has an algorithm to identify Asian surnames. MPRDR does not appear to contain any race and/or ethnicity information. Both PopData BC and MPRDR’s data holdings can be linked to the census to obtain area-level race and/or ethnicity data. 

## Discussion

Sample frames of surveys in Canada and its peer countries generally included the noninstitutionalized population of the country. The major concern of this strategy is that Black people are overrepresented in some institutions such as correctional institutions and in child welfare; therefore, excluding institutionalized populations may lead not only to greater exclusion of Black people, but also Indigenous and other racialized people in Canada. Black men are overrepresented in the federal prison populations, as 9% of inmates in 2020 were Black men, although they made up only 4.3% of the Canadian population in the 2021 Census.^18^,^61^ Moreover, these populations represent individuals with important health and social experiences that are not being captured routinely, such as the differential health impacts of incarceration or the child welfare system.

US surveys generally attempt to collect sufficient samples of non-White groups in their sampling strategies. For the most part, this is done by using area-level census data to identify areas that have high proportions of households with non-White residents, then oversampling in the strata with higher proportions of non-White residents. This strategy was abandoned by the NHIS in 2016, in favour of simple geographic representation,^62^ with an explanation that this new strategy might yield insufficient samples of non-White groups due to low response rates. Even if these groups were sampled in proportion to their populations, it could lead to small samples for small populations. In other words, locating and then oversampling these groups is critical to enable analysis of racial health inequities, provided that response rates can be improved. 

Canadian health surveys also mainly use area-level strategies to collect their samples, but they do not typically incorporate stratification of areas by racial and/or ethnic density of residents or conduct oversampling of non-White groups. This lack of appreciation for the importance of appropriate race-based data collection and usage is part of a wider problem in Canadian healthcare, identified in a recent report on race-based and Indigenous identity data collection.^63^ Given the availability of area-level information on racial and/or ethnic density through the long-form census, it is technically feasible to add this stratification and oversampling strategy to the current simple area-level strategy. In fact, when the HSE focussed on race and/or ethnicity, it applied area-level identification and oversampling of non-White groups. Moreover, the CHSS oversampled the population of Canadians aged 65 and older by using a telephone sampling frame to locate phone numbers of households with at least one senior. 

Some surveys in the US and New Zealand used administrative databases that contained information on race and/or ethnicity as sampling frames to locate non-White people. The US used this strategy for PRAMS, using birth records as the sampling frame, and New Zealand used this strategy for the NZHS, by using electoral rolls to locate Māori. In Canada, two administrative databases containing information on race and/or ethnicity are the long-form census and the landed immigrant registry (used as the sampling frame for the LSIC).† The long-form census contains data on race and/or ethnicity, and the landed immigrant registry contains information on country of origin, which might be used to approximate race and/or ethnicity. However, this approach would not be appropriate for estimating the race and/or ethnicity of newcomers from countries and regions that already have high levels of immigration, such as Europe or the US. 

So far in Canada, administrative records have been used as sampling frames for at least two surveys. The CSD obtains its sample from those who reported having limitations in activities of daily living on the Canadian long-form census in 2016 (for the 2017 CSD) and 2011 (for the 2012 CSD). The LSIC obtains its sample from the administrative database of all landed immigrants to Canada, provided by Immigration, Refugees and Citizenship Canada (formerly Citizenship and Immigration Canada).

An outlier in the data sources we reviewed was California’s CHIS, which recently began using a novel predictive modelling methodology to obtain samples of smaller racial and/or ethnic subgroups that might not be captured through area-based sampling. The strategy used many available datasets to predict the characteristics of households with residents who belong to specific, small, non-White groups. Once located through predictive modelling, these households were placed in a separate stratum, which was then oversampled. It is unclear whether this strategy is applicable in Canada, but it is worth exploring.

Administrative sources of health data differed significantly between Canada and some of its peer countries, because other countries (US and New Zealand) tend to have race and/or ethnicity contained within the record, and so no linkage is needed to match individuals’ health records to their race and/or ethnicity. New Zealand is the gold standard, where ethnicity is tied by law to a national health number and collected during various medical encounters. 

By contrast, in Canada, delivery and administration of health services and their datasets is the responsibility of provincial and territorial governments, rather than the federal government,‡ and race and/or ethnicity data for administrative health records can only be obtained through data linkages (e.g. Canadian long-form census and CCHS). There are limitations to this linkage, however. The long-form census is only administered every five years, so race and/or ethnicity data is only available every five years—meaning racial and/or ethnic information on individuals who were not eligible to complete the long-form census or who did not complete the census is missing. Since the long-form census is only administered to 25% of households, the remaining 75% are not queried about their race or ethnicity—representing a substantial data gap. Adopting pan-Canadian data collection standards is becoming more of a priority, given the important role that appropriate data collection plays in promoting equity.

***Strengths and limitations***

This review covered an extensive range of topics and considerations, and used in-depth methods to mitigate any omissions. We consulted not only peer-reviewed literature, but also guidance documents written by data administrators and government agencies provided for researchers using the datasets. 

The scope of this study was limited to health-related data sources in a few liberal welfare states to facilitate a timely review of data collection strategies and holdings. Other countries likely have additional or unique methods for routinely collecting information on sociodemographic characteristics that could have been informative for this review. Moreover, given our focus on health datasets, we did not identify insights into the collection and aggregation of race-based data in other socioeconomic surveys or datasets. 

A final limitation is that, because our analysis was chiefly focussed on identifying and comparing the methods employed by surveys and datasets, we did not thoroughly explore the ramifications of these approaches in practice, specifically, the question of whether or not race-based data collection actually captures the perceptions of race that can lead to adverse experiences among racialized individuals. There is a risk that the self-identified race and/or ethnicity data collected by these sources may not be consistent with how people are perceived—a critical factor in how people are treated, and therefore an important social determinant of health status. Most peer country data sources do not contain direct questions about discrimination or racism, and so they may be missing information on this direct pathway through which the health status of racialized individuals is impacted. This issue was explored in a recent Canadian study, in which the authors recommended that an additional question to the effect of: “Are you perceived or treated as a person of colour?” be added to questionnaires to assess whether individuals were experiencing racialization and racial discrimination.^66^

Statistics Canada’s General Social Survey also contains data on discrimination, but has far less information on health status, excluding it from the scope of our analysis. Research from it suggests that compared to White people, Black and Indigenous people in Canada experience routinely, and at far higher levels, being treated as not intelligent, receiving poor service in restaurants and stores, being feared by others and encountering other forms of “everyday discrimination” compared to other racial groups in Canada.^67^ There is also some evidence that these experiences of discrimination are associated with chronic health conditions.^67^ However, given that some forms of racism (such as structural racism) may not be as easily identified through questions like these, there have recently been calls in the literature to find ways to measure structural forms of racism, rather than “only” individual-level experiences of racism.^68^,^69^ We are not able to adequately address these issues in this report. 

Notwithstanding these limitations, we believe that the promising practices identified here can be feasibly implemented in Canada, and thereby strengthen data collection to support the study of racial and/or ethnic health inequities. Canadian health surveys should integrate targeted and oversampling strategies to ensure larger, more racially representative samples. Additionally, exploring predictive modelling techniques and incorporating race data directly into administrative sources, such as personal health card information, could enhance the representativeness and utility of health data. 

†“Landed immigrants” is a term used in the LSIC, which predated the establishment of the legal term “permanent resident” in 2001. Both terms refer to persons who have been granted the right to permanently live in Canada, but who have not yet become Canadian citizens.‡The exception to this is the health services of First Nations people living on reserves, Inuit, serving members of the Canadian Armed Forces, eligible veterans, inmates in federal penitentiaries and some refugee claimants, which are delivered and administered by the federal government.

## Conclusion

Canada’s ability to be informed about its racial health inequities lags behind some of its peer countries, primarily the US, New Zealand and Australia, but there are promising practices to be gleaned from these countries. Canada has fairly limited collection of data on race, particularly among administrative sources of data. In the short term, we recommend continuing to expand data linkages with sources that do contain race data (e.g. the long-form census, CCHS), which will increase capacity for analysis of racial health inequities. 

To facilitate high-quality research into racial health inequities, we strongly recommend encouraging these data linkages, and additionally, providing researchers with greater detail on the classification of specific racial and/or ethnic groups (while maintaining censorship over small samples). Given the prominent role that racism and discrimination play as social determinants of health, we also recommend that population-level health surveys ask respondents more questions about experiences of racism and discrimination to further this area of research. In the longer term, there must be serious conversations about implementing the routine collection and linkage of race-based data in administrative datasets, such as linking with the long-form census or the use of algorithms to identify race. 

Canada’s surveys currently do not have mechanisms for capturing large, representative samples of various racial groups. Based on our analysis, we found that Canadian health surveys also mainly use area-level strategies to collect their samples, but they rarely collect sufficient samples of non-White racial and/or ethnic groups. Given the availability of area-level information on racial and/or ethnic density in the census, it seems very feasible to add this stratification and oversampling strategy to the current simple area-level strategy that is being used. In the short term, we would recommend increasing these samples by revising the sampling strategy to include strata that are divided by area-level indicators of race and/or ethnicity, and to oversample from areas with higher levels of Black and other non-White groups. This will also allow for the implementation of more novel predictive modelling methodology (such as that used in the California CHIS) to improve sampling of these populations.

Although many of our data sources and approaches were discussed with a view to improving data collection and analysis of health inequities among Black Canadians, many groups can benefit from this research, including Indigenous folks and other undersampled populations in Canada. There are innovative and feasible methods, demonstrated by peer countries, that can strengthen the collection and usability of race-based data in Canada, ultimately leading to a more inclusive data infrastructure and enabling a more comprehensive understanding of health inequities and evidence-based policy solutions.

## Acknowledgements

This research was conducted jointly by PHAC employees, and by an external consultant funded by PHAC. 

## Conflicts of interest

None of the authors has any conflicts of interest to declare. 

## Authors’ contributions and statement

BJ, AS: conceptualization.

AS: funding acquisition.

MJ: investigation.

MJ, BJ, AS: methodology.

MJ, AB, BJ, AS: project administration.

AS: supervision.

MJ, AS: writing—original draft.

MJ, AB, BJ, AS: writing—review and editing.

The content and views expressed in this article are those of the authors and do not necessarily reflect those of the Government of Canada.
